# Integrated Molecular Docking and Molecular Dynamics Approaches Reveal Potential Antimicrobial Metabolites from Echinoderms, *Holothuria scabra*

**DOI:** 10.3390/biology15151259

**Published:** 2026-07-31

**Authors:** Merfat O. Aljhdli, Mohammad Habibur Rahman Molla, Saleh M. Al-Maaqar, Mohammed Othman Aljahdali

**Affiliations:** 1 Department of Chemistry, College of Science and Arts, King Abdulaziz University, Rabigh 21911, Saudi Arabia; moaljhdli@kau.edu.sa; 2 Department of Biological Sciences, Faculty of Science, King Abdulaziz University, Jeddah 21598, Saudi Arabia; saky7009@gmail.com (M.H.R.M.); salehalmagr@gmail.com (S.M.A.-M.); 3 Rubenstein School of Environment and Natural Resources, University of Vermont, Burlington, VT 05405, USA

**Keywords:** *H. scabra*, antimicrobial compounds, marine natural products, GC–MS, molecular docking, ADME, molecular dynamics simulation

## Abstract

The rapid emergence of multidrug-resistant microbial pathogens has created an urgent need for new antimicrobial agents. Marine organisms are a rich source of structurally diverse bioactive metabolites with therapeutic potential, and the sea cucumber *H. scabra* contains numerous secondary metabolites that may exhibit favorable predicted interactions with antimicrobial targets. This study aimed to identify potential antimicrobial metabolites from *H. scabra* using an integrated computational approach. Metabolites were identified by gas chromatography–mass spectrometry (GC–MS) and screened against selected bacterial and fungal target proteins using molecular docking. The top-ranked compounds were further evaluated for pharmacokinetic properties through ADME prediction, while the stability of the protein–ligand complexes was assessed using 100 ns molecular dynamics simulations and MM/GBSA binding free energy calculations. GC–MS analysis tentatively identified sixteen metabolites, among which CID 21820889 and CID 304 exhibited favorable predicted binding interactions with target proteins from *Acinetobacter baumannii* and *Candida albicans*, respectively. These compounds also demonstrated acceptable predicted pharmacokinetic properties, stable protein–ligand interactions throughout the simulations, and favorable MM/GBSA binding free energies, supporting the stability of the predicted complexes. Overall, MM/GBSA binding free energies ranged from −35.46 to −47.17 kcal/mol, with CID 441130 exhibiting the most favorable overall binding energy (−47.17 kcal/mol), while CID 21820889 also showed a favorable binding free energy (−44.85 kcal/mol). These findings identify several putatively identified, computationally prioritized metabolites from *H. scabra* as candidates for further investigation and demonstrate the utility of integrating chemical profiling with computational approaches for the early stage screening of marine natural products.

## Simple Summary

Antimicrobial resistance is reducing the effectiveness of many existing drugs, creating an urgent need for new treatments. Marine organisms are rich sources of natural compounds with potential medicinal value. In this study, the sea cucumber *Holothuria scabra* was investigated as a source of bioactive metabolites to identify compounds with potential antimicrobial activity. Chemical analysis detected sixteen metabolites, which were evaluated using computational methods to predict their interactions with proteins from harmful bacteria and fungi. Two compounds showed strong binding, favorable drug-like properties, and stable interactions during computer simulations. Although experimental validation is still required, these findings suggest that *H. scabra* is a promising source of natural antimicrobial compounds and demonstrate the value of computational approaches for accelerating marine drug discovery.

## 1. Introduction

Antimicrobial resistance (AMR) has emerged as one of the most pressing global public health challenges, threatening the effectiveness of existing antibiotics and antifungal agents [1]. The rapid dissemination of multidrug-resistant (MDR) pathogens, coupled with the limited development of new antimicrobial drugs, has created an urgent need to identify novel therapeutic agents with unique mechanisms of action [2]. Marine organisms have become increasingly attractive sources of structurally diverse bioactive metabolites owing to their adaptation to highly competitive and dynamic environments [3]. Among marine invertebrates, sea cucumbers (Holothuroidea) represent a particularly valuable reservoir of secondary metabolites with diverse pharmacological activities, including antimicrobial, antioxidant, anti-inflammatory, anticancer, antiviral, and immunomodulatory properties [4].

*H. scabra* (sandfish) is one of the most commercially and ecologically important sea cucumber species distributed throughout the Indo-Pacific region, including the Red Sea coast of Saudi Arabia [5]. Beyond its ecological role as an ecosystem engineer that promotes sediment bioturbation, nutrient recycling, and benthic ecosystem health, *H. scabra* is highly valued in traditional medicine and the seafood industry [6]. Phytochemical investigations have demonstrated that *H. scabra* contains numerous bioactive constituents, including triterpene glycosides (saponins), sulfated polysaccharides, peptides, fatty acids, sterols, and other secondary metabolites with considerable pharmaceutical potential [7]. Several studies have reported antimicrobial activity of crude extracts against both bacterial and fungal pathogens; however, the specific compounds responsible for these activities and their molecular mechanisms remain largely unexplored [8].

Among clinically important MDR pathogens, *A. baumannii* has become one of the most problematic causes of healthcare-associated infections worldwide [9]. This opportunistic Gram-negative bacterium exhibits remarkable resistance to multiple classes of antibiotics through mechanisms including β-lactamase production, biofilm formation, efflux pumps, and horizontal acquisition of resistance genes [10]. Consequently, infections caused by *A. baumannii* are associated with prolonged hospitalization, increased healthcare costs, and high mortality rates [11]. Likewise, *C. albicans* remains the leading opportunistic fungal pathogen responsible for superficial and invasive candidiasis, particularly in immunocompromised individuals [12]. The increasing prevalence of antifungal resistance and biofilm-associated infections further limits available therapeutic options, emphasizing the need for new antifungal agents [13].

Structure-based drug discovery provides an efficient strategy for identifying natural compounds capable of inhibiting essential virulence-associated proteins in these pathogens [14]. The class C β-lactamase of *A. baumannii* (PDB ID: 6WIF) is a validated antibacterial target because it hydrolyzes β-lactam antibiotics, thereby conferring resistance to cephalosporins and related antimicrobial agents [15]. Similarly, Secreted Aspartyl Proteinase 2 (Sap2) of *C. albicans* (PDB ID: 1ZAP) is a major virulence factor involved in host tissue invasion, nutrient acquisition, biofilm formation, and immune evasion, making it an attractive target for antifungal drug development [16]. The availability of high-resolution crystal structures for both proteins enables detailed investigation of ligand-binding mechanisms using computational approaches [17].

Recent advances in computer-aided drug design (CADD) have significantly accelerated the discovery of bioactive compounds from natural products by integrating molecular docking, molecular dynamics (MD) simulations, and binding free-energy calculations [18]. These computational approaches provide valuable insights into ligand–protein interactions, conformational stability, and binding mechanisms while reducing the time and cost associated with experimental screening [19]. The integration of these in silico techniques offers an efficient and reliable strategy for identifying promising antimicrobial lead compounds from chemically diverse marine natural products [20].

The present study investigated the antimicrobial potential of bioactive compounds identified from *H. scabra* using an integrated computer-aided drug discovery workflow [21]. Chemical constituents of *H. scabra* extract were identified by gas chromatography–mass spectrometry (GC–MS), and selected compounds were subsequently evaluated against the class C β-lactamase of Acinetobacter baumannii (PDB ID: 6WIF) and Secreted Aspartyl Proteinase 2 (Sap2) of Candida albicans (PDB ID: 1ZAP) using molecular docking, molecular dynamics simulations, and MM/GBSA binding free-energy analyses. This integrated computational approach provides molecular-level insights into the interactions between *H. scabra*-derived metabolites and key antimicrobial targets, facilitating the identification of promising lead compounds for the development of novel antibacterial and antifungal agents.

## 2. Materials and Methods

### 2.1. Sample Collection and Preparation of H. scabra

Adult specimens of *Holothuria scabra* were collected from the Red Sea coast near Jeddah, Saudi Arabia, during the summer season by SCUBA diving. Specimens were taxonomically identified based on external morphological characteristics using standard identification keys by marine taxonomists in the Department of Biological Sciences, King Abdulaziz University. Following collection, the samples were transported to the laboratory on ice and thoroughly rinsed with distilled water to remove adhering sand, epibionts, and other debris. The visceral tissues were carefully dissected, air-dried at room temperature until a constant weight was achieved, and ground into a fine powder using a laboratory grinder (IKA-Werke GmbH & Co. KG, Staufen, Germany). Dried tissue powder (100 g) was extracted with 500 mL of 95% ethanol (1:5, *w*/*v*) (Merck KGaA, Darmstadt, Germany) by maceration at room temperature (25 ± 2 °C) for 72 h with intermittent agitation. The extraction was repeated three times to ensure maximum recovery of bioactive metabolites. The combined extracts were filtered through Whatman No. 1 filter paper (Cytiva, Marlborough, MA, USA) and concentrated under reduced pressure using a rotary evaporator (Buchi Rotavapor R-114 & Waterbath B-480, Buchi Labortechnik AG, Flawil, Switzerland) at 40 °C. The resulting crude ethanolic extract was stored at 4 °C in airtight containers until subsequent GC–MS analysis and antimicrobial assays.

### 2.2. Compound Isolation (Gas Chromatography–Mass Spectrometry)

The chemical constituents of the ethanolic extract of *H. scabra* were analyzed using gas chromatography–mass spectrometry (GC–MS) equipped with a capillary column (Agilent Technologies, Santa Clara, CA, USA). Briefly, 100 g of dried powdered *H. scabra* tissue was macerated in 500 mL of 95% ethanol at room temperature, and the crude extract was concentrated using a rotary evaporator (Buchi Rotavapor R-114 & Waterbath B-480, Switzerland) prior to GC–MS analysis. GC–MS analysis was performed using an Agilent 7890B gas chromatograph coupled with an Agilent 5977A Mass Selective Detector (Agilent Technologies, Santa Clara, CA, USA), equipped with an HP-5MS fused silica capillary column (5% phenyl methylpolysiloxane; 30 m × 0.25 mm i.d. × 0.25 μm film thickness) (Agilent Technologies, Santa Clara, CA, USA). An aliquot of 1 μL of the extract was injected in split mode (split ratio 10:1). Helium (99.99% purity) was used as the carrier gas at a constant flow rate of 1.0 mL min^−1^. The oven temperature program was set as follows: the initial temperature was maintained at 50 °C for 1 min, increased to 200 °C at a rate of 10 °C min^−1^, and then further increased to 300 °C, where it was maintained until the end of the analysis. The total run time was 45 min. The injector temperature was maintained at 280 °C. The mass spectrometer (Agilent Technologies, Santa Clara, CA, USA). was operated in electron ionization (EI) mode at 70 eV, with a mass scan range of *m*/*z* 40–600 in full-scan mode. Compounds were identified by comparing their mass spectra with those available in the National Institute of Standards and Technology (NIST) Mass Spectral Library and by comparing their retention times with published literature where applicable [5].

### 2.3. Protein Preparation, Refinement, and Validation

The three-dimensional structure of the *A. baumannii* protein was retrieved from the RCSB Protein Data Bank (PDB) using the PDB ID 6WIF (Class C beta-lactamase from *A. baumannii* in complex with 4-(Ethyl(methyl)carbamoyl)phenyl boronic acid), while the structure of the *C. albicans* target protein was obtained under the PDB ID 1ZAP (Secreted aspartic protase). Both protein structures were downloaded in PDB format and subsequently refined using the GalaxyRefine server (http://galaxy.seoklab.org/cgi-bin/submit.cgi?type=REFINE, accessed on 25 January 2025) to improve structural quality prior to molecular docking. Structural validation was performed using Ramachandran plot analysis to assess the distribution of backbone dihedral angles and confirm the reliability of the modeled structures. For docking preparation, all metal ions, water molecules, and cofactors were removed to avoid interference with ligand binding. Additionally, Gasteiger charges were calculated, and polar hydrogens were added, while non-polar hydrogens were merged to ensure accurate simulation of molecular interactions during docking. These refined and validated protein models were then used for molecular docking studies to predict the binding affinity and interaction profiles of sea cucumber-derived bioactive compounds [22]. The identification of suitable target proteins was based on their biological relevance to the pathogenicity and drug resistance mechanisms of the selected microorganisms. For *A. baumannii*, the target protein used for molecular docking was retrieved from the RCSB Protein Data Bank (PDB ID: 6WIF).

### 2.4. Documented Active Sites and Receptor Grid Generation

**Figure 1:**
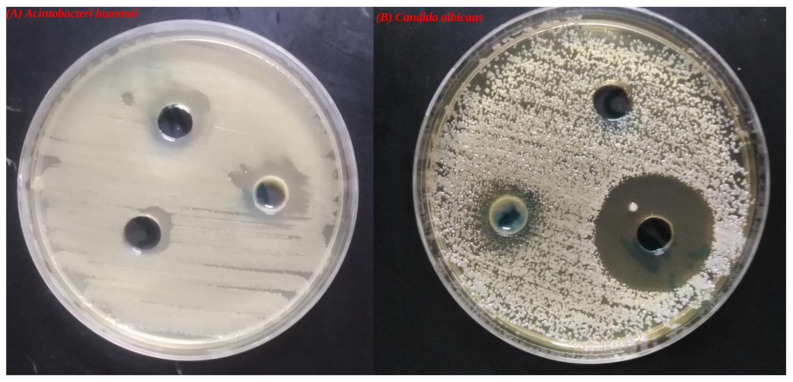
Confirmation of inhibitory activity of CID 21820889 against *A. baumannii* (**A**) and CID 304 against *C. albicans* (**B**).

The active site (AS) of an enzyme is a specialized region formed by the spatial arrangement of specific amino acid residues capable of forming temporary interactions with the substrate, known as the binding site. This region enables the enzyme to bind its substrate and catalyze the biochemical reaction while stabilizing reaction intermediates. The binding site represents the portion of a protein or nucleic acid that recognizes a ligand and forms a stable interaction with it. The active site of each target protein was initially predicted, and the binding-site residues were subsequently identified to define the docking region. Analysis of the 1ZAP protein revealed several key residues forming the active pocket (Figure 1A). The identified binding site residues were located at ASN9, GLN11, THR13, ILE30, THR50, PHE58, TYR84, SER118, PRO162, ARG195, LEU216, ASP245, SER277, THR283, GLY285, GLN295, ASP299, ILE305, and ARG312, represented in different colors in Figure 1A. Further pocket analysis of the 6WIF protein identified additional residues contributing to the binding site, including PRO336, ARG370, MET359, LEU43, ARG364, GLN229, and LYS363 (Figure 1B). These identified residues were then used to construct receptor grids for molecular docking simulations. The grid box dimensions were set to X = 93.1, Y = 52.14, and Z = 43.2 Å for 1ZAP protein, and X = 38.07, Y = 25.95, and Z = 33.87 Å for 6WIF protein, as shown in Figure 1A,B [23].

### 2.5. Molecular Docking Between H. scabra Compounds and Receptor

Molecular docking analysis was performed using the 16 bioactive compounds identified from *H. scabra* against the selected target protein receptors to identify compounds with the highest binding affinity and potential inhibitory activity. The PyRx program (version 0.8) was employed for molecular docking, and the AutoDockVina tool (version 1.2.0) was used to validate the docking scores and interactions between the ligands and the receptor [24]. Active site residues interacting with ligands were identified using PyMOL (version 2.5.5) and LigPlot+ (version 2.2). The chemical classes and structural scaffolds of lead compounds were determined using PubChem and relevant literature. The RMSF analysis was performed to evaluate residue flexibility, including N-terminal, C-terminal, and active site regions, highlighting regions of high atomic fluctuation and structural stabilization upon ligand binding.

### 2.6. Molecular Descriptor Calculation

The chemical structures of the sixteen compounds identified through GC–MS analysis were retrieved from the PubChem database in SDF format. Molecular descriptors representing physicochemical and structural properties of each compound were calculated using the PaDEL-Descriptor software (version 2.21). These descriptors included molecular weight, hydrogen-bond donors and acceptors, topological polar surface area (TPSA), logP, rotatable bonds, aromatic ring counts, and several topological indices. A total of more than 100 molecular descriptors were initially generated for each compound. Prior to model training, the dataset was preprocessed to remove redundant or highly correlated descriptors using a Pearson correlation threshold of 0.90. The remaining descriptors were normalized to ensure consistent scaling across variables.

#### Prediction of Antimicrobial Activity

Following the molecular docking and ADME analyses, the sixteen compounds identified from *H. scabra* were comparatively evaluated based on their binding affinities, protein–ligand interaction profiles, and predicted pharmacokinetic properties. Compounds exhibiting favorable docking scores together with acceptable ADME characteristics were prioritized as promising candidates for subsequent molecular dynamics simulations and further experimental investigation.

### 2.7. ADME and Toxicity Analysis

In drug discovery, the selection of the right molecules is crucial to minimize in vivo and in vitro failure rates. The Swiss-ADME web-based system (http://www.swissadme.ch/, accessed on 25 January 2025) was used to assess the bioavailability and solubility of promising compounds. Additionally, their safety profiles were evaluated using CADD methods to determine potential toxicity risks. Toxicological evaluations included assessments of mutagenicity, carcinogenicity, LD50 values, and immunotoxicity, which were conducted using the ProTox-II web server (http://tox.charite.de/protox_II, accessed on 25 January 2025) [25].

### 2.8. Molecular Dynamics (MD) Simulation

To further evaluate the dynamic stability of the protein–ligand complexes obtained from molecular docking, 100 ns molecular dynamics (MD) simulations were performed using the Desmond Molecular Dynamics System implemented in Schrödinger Release 2023-2 (Schrödinger, LLC, New York, NY, USA). The top-ranked docking pose of each protein–ligand complex was selected as the initial structure for the simulation. The systems were prepared using the Protein Preparation Wizard and simulated with the OPLS4 force field. Each complex was solvated in an explicit TIP3P water model within an orthorhombic simulation box with a 10 Å buffer distance between the protein complex and the box boundaries. Periodic boundary conditions were applied in all three spatial dimensions. The systems were electrically neutralized by adding appropriate Na^+^ counterions, and 0.15 M NaCl was added to mimic physiological ionic strength. Before the production simulation, each system underwent energy minimization followed by the default Desmond relaxation protocol to remove unfavorable steric contacts and equilibrate the solvent environment. Production MD simulations were performed for 100 ns under the isothermal–isobaric (NPT) ensemble at 300 K and 1.01325 bar. Temperature and pressure were maintained using the Nose–Hoover thermostat and the Martyna–Tobias–Klein barostat, (Schrödinger, LLC, New York, NY, USA) respectively. Simulation trajectories and energy data were recorded at 50 ps intervals for subsequent analyses [26].

### 2.9. Trajectory Analysis of MD Simulation

The MD trajectories were analyzed using the Simulation Interaction Diagram (SID) module (Schrödinger Release 2023-2) implemented in Schrödinger Maestro (Schrödinger Release 2023-2) to evaluate the structural stability and dynamic behavior of the protein–ligand complexes throughout the 100 ns simulation period. Structural stability was assessed by calculating the root mean square deviation (RMSD) of the protein backbone and ligand, whereas residue-level flexibility was evaluated using the root mean square fluctuation (RMSF). The overall compactness of each protein was monitored through the radius of gyration (Rg) analysis, while the stability of protein–ligand interactions was assessed by monitoring the number and persistence of hydrogen bonds formed during the simulation [27].

### 2.10. RMSD Evaluation

RMSD was calculated to assess the structural stability of the protein–ligand complexes during the simulation. It measures the average displacement of atoms in the complex over time relative to a reference structure. After aligning the protein backbones, RMSD values were tracked across the full 100 ns simulation period to monitor conformational changes and overall system stability [28].

#### RMSF Evaluation

RMSF analysis was employed to evaluate local flexibility in the protein structure. It measures the fluctuation of each residue or atom around its average position during the simulation. This metric helps in identifying regions of the protein that undergo significant conformational variation upon ligand binding, providing insight into dynamic changes at the binding site [29].

### 2.11. Solvent Accessible Surface Area (SASA) Analysis

The solvent accessible surface area (SASA) was calculated throughout the molecular dynamics (MD) simulations to evaluate the degree of solvent exposure and structural compactness of the protein–ligand complexes. SASA represents the surface area of the protein accessible to a solvent probe and is widely used to monitor conformational changes during MD simulations. Lower SASA values generally indicate a more compact protein structure with reduced solvent exposure, whereas higher SASA values suggest structural expansion or increased flexibility. SASA calculations were performed using the trajectory analysis module of the MD simulation package (Desmond, Release 2023-2, Schrödinger, LLC, New York, NY, USA), with a standard solvent probe radius of 1.4 Å. The SASA values were recorded at regular intervals over the simulation. Histograms of SASA distributions were also generated to evaluate the frequency of sampled conformational states.

### 2.12. MM/GBSA Binding Free Energy Calculation

To further evaluate the relative binding affinities and energetic stability of the protein–ligand complexes, Molecular Mechanics Generalized Born Surface Area (MM/GBSA) binding free energy calculations were performed using the Prime MM/GBSA module (Release 2023-2, Schrödinger, LLC, New York, NY, USA) implemented in the Schrödinger Maestro suite (Schrödinger LLC, New York, NY, USA). Representative snapshots extracted from the equilibrated region of the 100 ns molecular dynamics (MD) trajectories generated with Desmond were used for the calculations. The same force field employed during the MD simulations (OPLS_2005) was retained throughout the MM/GBSA calculations to ensure methodological consistency. The MM/GBSA calculations decompose the binding free energy into multiple energetic components to provide insights into the driving forces governing protein–ligand interactions. In this study, the following energy descriptors were analyzed: MMGBSA ΔG-binding energy, representing the overall predicted binding free energy of the complex; MMGBSA ΔG-bind in Coulomb, corresponding to the electrostatic contribution to binding; MMGBSA ΔG-bind (NS), representing the non-solvated interaction energy; and MMGBSA ΔG-bind (NS)-Coulomb, representing the electrostatic contribution under non-solvated conditions.

### 2.13. Preparation of Crude Extracts

Sea cucumber samples were first dried and then finely ground using an electric blender (Panasonic Corporation, Osaka, Japan). The powdered material was placed in a glass flask containing 400 mL of 80% ethanol and subjected to continuous shaking at 150 rpm for 48 h at room temperature using an SHO 1-D shaker (Daihan Scientific Co., Ltd., Wonju, Republic of Korea). After incubation, the mixture was filtered to remove solid residues. The filtrate was then concentrated at 55 °C under reduced pressure using a rotary evaporator (Buchi Rotavapor R-114 & Waterbath B-480, Switzerland) to remove the solvent. The resulting dry extract was weighed using a high-precision electronic balance (ADAM Equipment Co., Ltd., Milton Keynes, UK; readability 0.0001 g), and the extraction yield was calculated. Finally, the extract was stored in a dark glass container at 4 °C until further use [30].

### 2.14. Pathogenic Bacteria and Fungal Strains

Two common pathogenic microorganisms used in this study included the fungal strain *C. albicans* ATCC 14053 and Gram-negative bacterium *A. baumannii* (MT875278). Both pathogenic microbes were obtained from the Department of Biology, Faculty of Science, King Abdulaziz University, Jeddah, Saudi Arabia.

### 2.15. Well Diffusion Method

The antibacterial activity of the test extract was evaluated using the agar well diffusion method. Bacterial cultures were prepared at a concentration of 10^6^ CFU/mL and evenly spread on Mueller–Hinton Agar (MHA) plates (Oxoid Ltd., Basingstoke, UK). Using a sterilized cork borer, three wells were created in each agar plate. One well was filled with sterile distilled water (SDW) to serve as a negative control, while the other two wells were loaded with 100 µL of the test extract at a concentration of 0.5 and 1 mg/mL. The plates were incubated at 37 °C for 24 h. Following incubation, the zones of inhibition around each well were measured. All experiments were performed in triplicate, and results were reported as mean ± standard deviation (SD). Data were analyzed using two-way analysis of variance (ANOVA) followed by Tukey’s post hoc test for multiple comparisons. All statistical analyses were performed with GraphPad Prism 9.5.0, and a *p*-value < 0.05 was considered statistically significant.

## 3. Results

### 3.1. Sea Cucumber Compound Isolation Through the GC–MS

The isolation of compounds from *H. scabra* was carried out using gas chromatography coupled with mass spectrometry (GC–MS). A total of 16 distinct compounds were successfully identified during the analysis of the *H. scabra* samples. The GC–MS method allowed for the precise recording of key parameters such as retention time, chemical formula, and peak area up to a retention period of 50 min (Figure S2). As detailed in Figure S1, the majority of the compounds were detected within the retention window between 38.82 and 43.07 min. These findings highlight the effectiveness of GC–MS in isolating and characterizing bioactive compounds from marine organisms such as *H. scabra* (Table 1).

### 3.2. In Vitro Experiment

Out of the 16 sea cucumber compounds tested for antimicrobial activity, two compounds, CID 21820889 and CID 304, showed significant efficacy against specific microbial pathogens. CID 21820889 exhibited potent activity against *A. baumannii*, a Gram-negative bacterium, while CID 304 demonstrated effectiveness against *C. albicans*, a fungal strain. Both compounds were tested using the well diffusion method, where they were applied at a concentration of 1 mg/mL in sterile extracts. For *A. baumannii*, CID 21820889 produced a noticeable zone of inhibition, indicating its potential as an effective therapeutic agent against this bacterial pathogen. Similarly, CID 304 showed a strong inhibitory effect against *C. albicans*, highlighting its antifungal potential. The computational analyses identified CID 21820889 and CID 304 as computationally prioritized candidate metabolites with favorable predicted interactions against the selected target proteins of *A. baumannii* and *C. albicans*, respectively (Figure 1A,B). In contrast, the minimum inhibitory concentration (MIC) and minimum bactericidal concentration (MBC) values reported in this study were determined for the crude ethanolic extract of *H. scabra* and should not be attributed to any individual GC–MS-putatively identified compound. The MIC values ranged from 0.5 to 10 mg/mL, and the MBC values ranged from 1 to >128 mg/mL, consistent with findings from other studies on *A. baumannii*. For *C. albicans*, the MIC and MBC values ranged from 0.25 to 1 µg/mL, aligning with reported ranges for antifungal agents (Figure 1).

**Figure 2:**
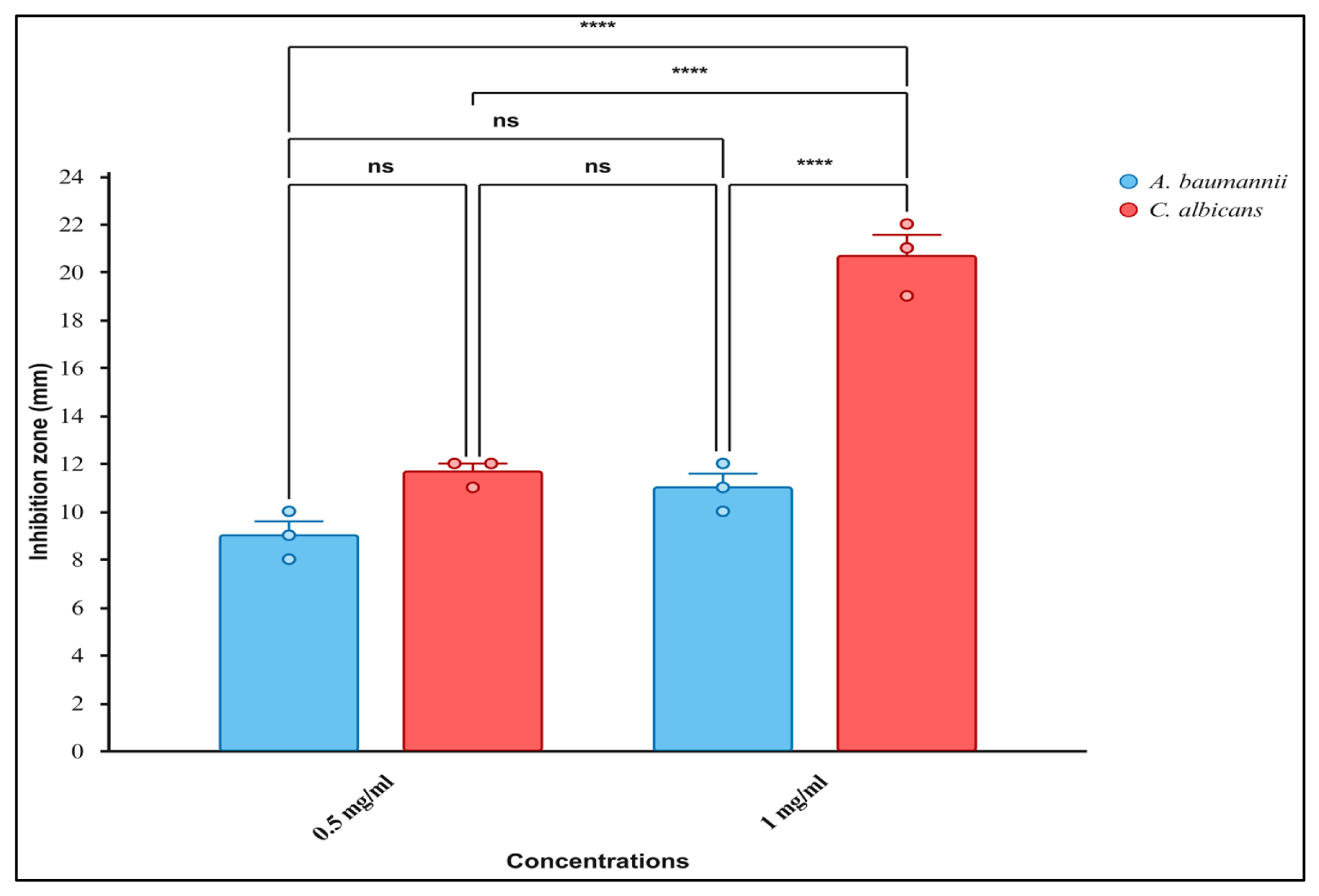
Antimicrobial activity of the crude ethanolic extract of *Holothuria scabra* against *Acinetobacter baumannii* and *Candida albicans* at concentrations of 0.5 and 1.0 mg/mL. Data are presented as mean ± SD (*n* = 3). Statistical significance was analyzed using two-way ANOVA followed by Tukey’s multiple comparisons test. “****” indicates *p* < 0.0001, while “ns” indicates not statistically significant (*p* > 0.05).

Based on a two-way ANOVA, there were significant main effects of pathogen (*p* < 0.0001) and concentration (*p* < 0.0001), along with a significant interaction *(p* = 0.0005) (Figure 2). Tukey’s post hoc tests showed that for *C. albicans*, increasing the concentration from 0.5 to 1 mg/mL significantly increased inhibition (from 11.67 to 20.67 mm, *p* < 0.0001), and both *C. albicans* groups outperformed their *A. baumannii* counterparts at 1 mg/mL (*p* < 0.0001). In contrast, *A. baumannii* showed no significant change with concentration (*p* = 0.185). Variance was homogeneous (Levene’s *p* = 0.728), supporting the ANOVA findings (Table S3).

### 3.3. Validation, Refinement and Receptor Preparation

The proteins were found in the well-known databank RCS-PDB, and the Ramachandran plot was used to figure out which areas were allowed and not allowed. To construct the protein, molecular docking methods were used, and the protein was kept in a pdbqt format for molecular docking. The Z-score evaluates the overall quality of a protein structure by comparing its energy profile with those of experimentally solved proteins of similar size. The Z-scores of 1ZAP (−8.3) and 6WIF (−9.92) fall within the range of native X-ray and NMR structures, indicating good structural quality and supporting their suitability for molecular docking and molecular dynamics analyses (Table S1). A Ramachandran plot of the 1ZAP protein was generated to assess the stereochemical quality of the protein structure. The blue contour lines indicate the favored and additionally allowed regions for the backbone φ (phi) and ψ (psi) dihedral angles, reflecting energetically permissible conformations of the amino acid residues. The majority of amino acid residues are located within the favored regions, indicating good stereochemical quality and structural reliability of the protein model. The Ramachandran plot of the 6WIF protein illustrates the distribution of backbone dihedral angles (φ and ψ) of amino acid residues. Blue contour lines represent the favored and additionally allowed conformational regions. Most residues are located within the energetically favored β-sheet and right-handed α-helical regions, whereas only a small number of residues occur outside these regions, confirming the high stereochemical quality and structural reliability of the 6WIF protein model (Figure S3A,B).

### 3.4. Phytochemical and Protein Preparation

Sixteen bioactive compounds were identified from the sea cucumber extract using GC-MS analysis. These compounds were retrieved and saved in 2D SDF format. During the ligand preparation phase, all compounds were processed and optimized, and then converted into the PDBQT format for molecular docking studies. The target protein structure was also refined and prepared using AutoDock Tools (version 0.8; PyRx Virtual Screening Tool), and subsequently saved in PDBQT format for docking simulations [31].

### 3.5. Active Site Identification and Receptor Grid Generation

**Figure 3:**
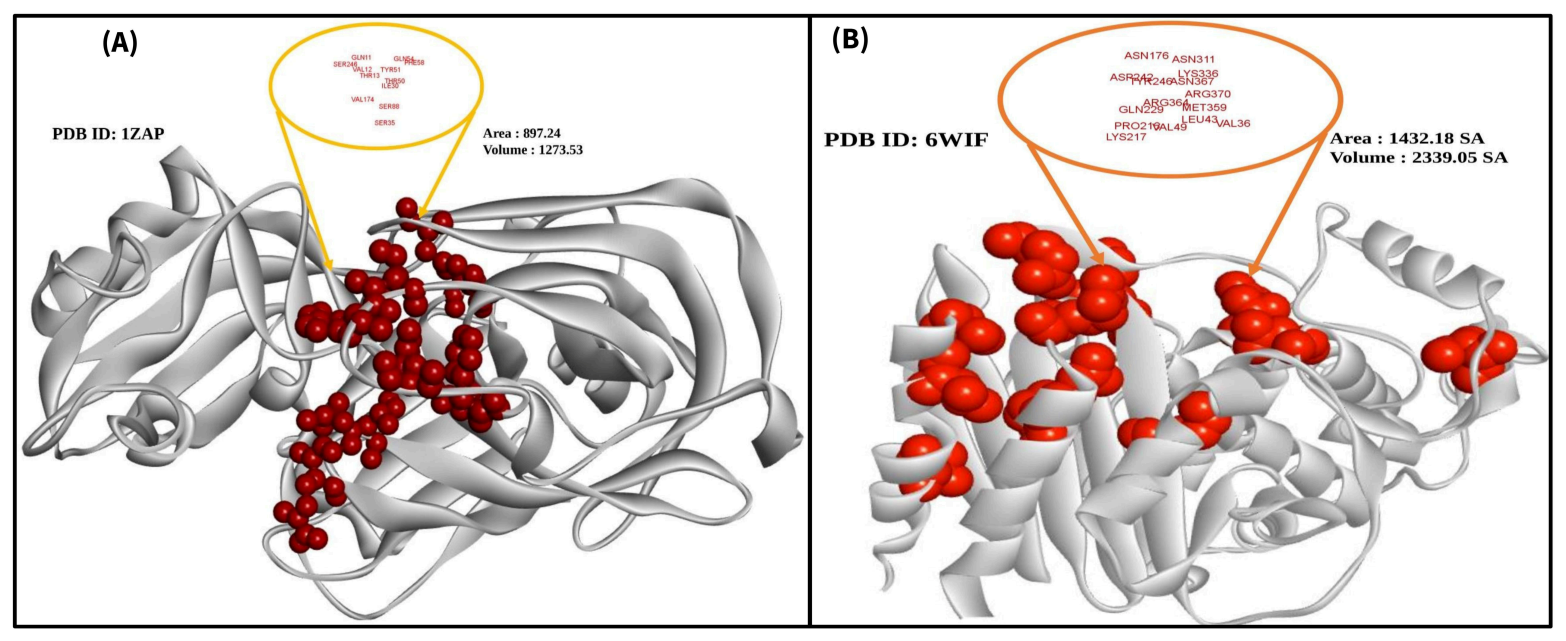
Active pocket showing two selected proteins: 1ZAP 1 (**A**) and 6WIF 1 (**B**).

The active site (AS) of an enzyme is formed by the specific arrangement of amino acid residues within a region of the protein, enabling the formation of temporary bonds with the substrate, known as the binding site. The AS of a protein facilitates the binding of a chemical substrate, leading to a catalyzed reaction, and also helps stabilize the reaction intermediates. The binding site refers to the specific area of a protein or nucleic acid that can recognize a ligand and form strong interactions with it. In this study, the AS of the gag polypeptide was identified using the CASTpi server, and the combined binding positions of the active site were retrieved (Figure 3). The analysis of the protein IZAP active pocket revealed key binding site residues (Figure 1A). The active site pocket analysis identified the binding site residues at positions ASN9, GLN11, THR13, ILE30, THR50, PHE58, TYR84, SER118, PRO162, ARG195, LEU216, ASP245, SER277, THR283, GLY285, GLN295, ASP299, ILE305, and ARG312, represented in ball-and-stick form with different colors, such as pink, as shown in Figure 3A. Further analysis of the active pocket of the protein 6WIF helped to retrieve additional binding site residues, which were found at positions PRO336,ARG370, MET359, LEU43, ARG364, GLN229, and LYS363 (Figure 3B).

### 3.6. Molecular Docking

Molecular docking was performed to evaluate the binding affinity of the sixteen bioactive compounds identified from the ethanolic extract of H. scabra against the selected antimicrobial target proteins from A. baumannii (PDB ID: 6WIF) and C. albicans (PDB ID: 1ZAP). The docking analysis identified several compounds with favorable binding affinities, indicating their potential as antimicrobial agents (Table 2). For the 6WIF target, the binding affinities ranged from −4.1 to −8.2 kcal/mol, with CID 21820889 exhibiting the strongest interaction (−8.2 kcal/mol), followed by CID 6432458 (−7.9 kcal/mol) and CID 304 (−6.7 kcal/mol). Similarly, for the 1ZAP target, binding affinities ranged from −4.4 to −8.4 kcal/mol, where CID 304 demonstrated the highest binding affinity (−8.4 kcal/mol), closely followed by CID 6432458 (−8.3 kcal/mol) and CID 21820889 (−7.7 kcal/mol). Notably, the selected compounds exhibited binding affinities comparable to or better than the reference drugs, which showed docking scores of −7.7 kcal/mol against 1ZAP, highlighting their potential as promising antimicrobial candidates.

Detailed interaction analysis further demonstrated that the selected compounds formed multiple stabilizing interactions within the active sites of the target proteins, including hydrogen bonds, hydrophobic interactions, and van der Waals contacts with key catalytic residues. These interactions are expected to enhance the stability of the protein–ligand complexes and may contribute to the observed high binding affinities, suggesting a potential inhibitory effect on the target proteins (Table 2).

### 3.7. Protein–Ligand Interaction Analysis for A. bummni

**Figure 4:**
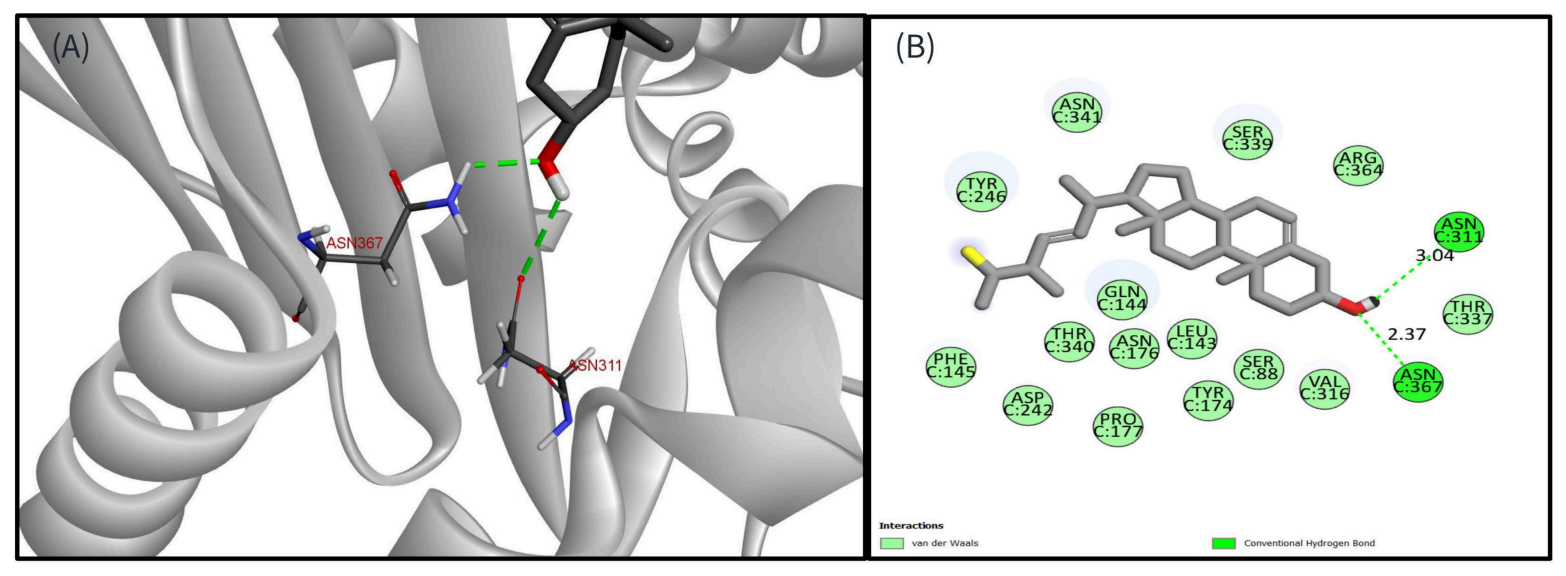
The 3D image displaying the interaction within receptor and compound CID 21820889 (**A**). The 2D image showing the interaction with different bonds (**B**).

The protein–ligand complex with the highest binding affinity was selected to analyze molecular interactions. Interactions between the chosen ligands and target proteins were visualized using the BIOVIA Discovery Studio Visualizer 16.1.0.41 [32]. For compound CID 21820889, both hydrogen bonding and hydrophobic interactions were identified with the target protein 6WIF. A hydrogen bond was observed at the ASN311 and ASN367 residue. These interactions are illustrated in Figure 4A,B.

### 3.8. Protein–Ligand Interaction Analysis for C. albicans

**Figure 5:**
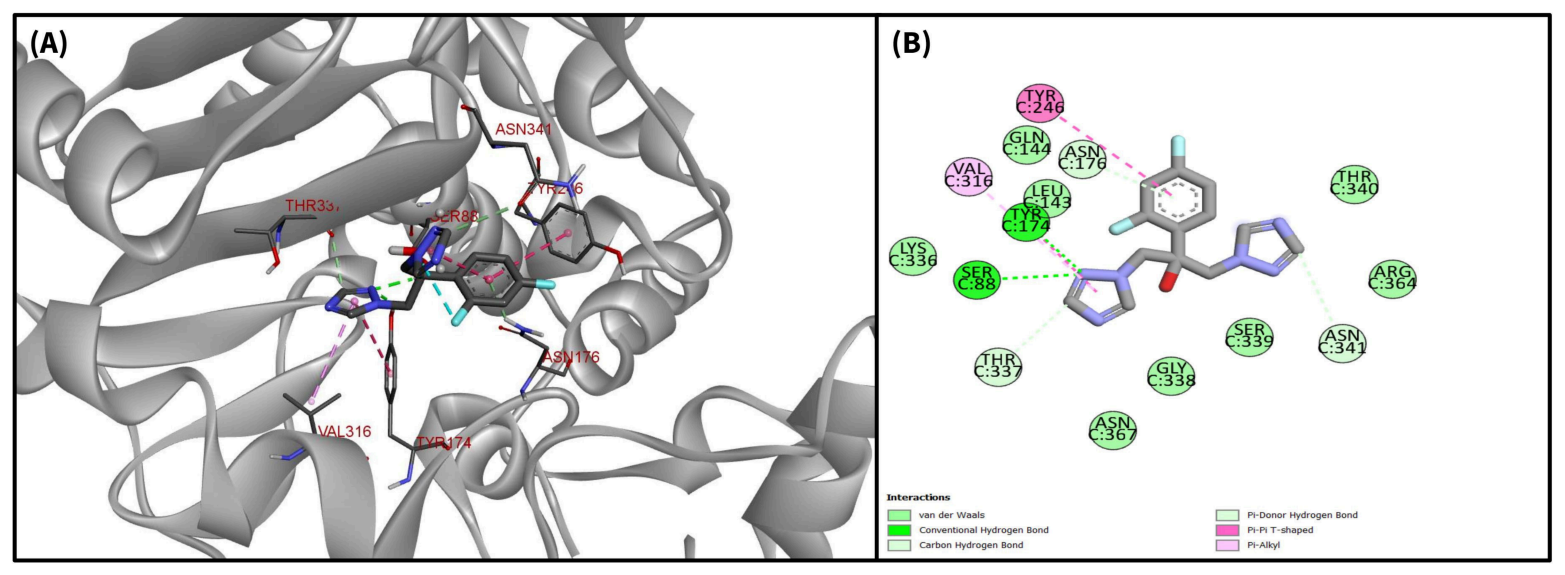
The 3D image displaying the interaction within receptor and compound CID 304 (**A**). The 2D image showing the interaction with different bonds (**B**).

The compound associated with the highest docking score was selected to evaluate its molecular interactions with the target protein. Visualization of the binding interactions between the three selected ligands and the receptor was carried out using the BIOVIA Discovery Studio Visualizer [33]. In the case of compound CID 304, the analysis revealed the presence of both hydrogen and hydrophobic interactions. A hydrogen bond was detected at the TYR174 and SER88 residue, while Pi-Pi T shaped contacts were observed at TYR246. These binding interactions are illustrated in Figure 5A,B.

### 3.9. ADME Analysis

Pharmacokinetics (PK), derived from the Greek words pharmakon (drug) and kinetikos (movement), plays a crucial role in the drug design and development process. It primarily focuses on ADME (absorption, distribution, metabolism, and excretion) properties, along with key physicochemical factors such as lipophilicity, water solubility, pharmacokinetics, drug likeness, and medicinal chemistry. These properties help form hypotheses for identifying the most promising drug candidates. Prior to preclinical studies, assessing pharmacophore properties is essential for understanding a compound’s interaction with xenobiotic regulation. The SwissADME server was utilized to analyze the pharmacophore properties of three selected drug-like compounds. These compounds exhibited lipophilicity, suggesting they are soluble in fats, oils, and nonpolar solvents. The pharmacophore analysis indicated that the compounds are suitable candidates for further study due to their potential effectiveness and druggable properties. The pharmacokinetics of these compounds are summarized in Table 3. The list of pharmacokinetics properties includes the ADME properties of the selected three compounds. The list also presents the different physicochemical properties of the three compounds.

In this study, two compounds, CID 21820889 and CID 304, were compared across various physicochemical and pharmacokinetic properties. Both compounds have similar molecular weights, with CID 21820889 weighing 378.42 g/mol and CID 304 at 386.65 g/mol. CID 21820889 is characterized by a higher number of aromatic heavy atoms (26), while CID 304 contains none. Notably, CID 21820889 has fewer rotatable bonds (3) compared to CID 304, which has five, suggesting a difference in molecular flexibility. The hydrogen-bonding profiles differ significantly, with CID 21820889 having more hydrogen-bond donors (1) and acceptors (3) than CID 304, which has only one of each. Regarding lipophilicity, CID 304 is more hydrophobic, with a higher Log Po/w of 7.39 compared to 4.94 for CID 21820889. This is reflected in the water solubility data, where CID 21820889 is more soluble (−5.47 Log S) than CID 304 (−7.40 Log S). Pharmacokinetically, CID 21820889 demonstrates high gastrointestinal absorption, while CID 304 exhibits low absorption, indicating potential differences in bioavailability. Both compounds violate Lipinski’s Rule of Five, which may suggest challenges in their oral bioavailability. Additionally, CID 304 has a higher synthetic accessibility score, implying it may be easier to synthesize than CID 21820889. These findings underscore important structural and functional differences between the two compounds, with implications for their potential drug development (Table 3).

### 3.10. Toxicity Prediction

Assessing the potential harmful effects of a compound on humans, animals, plants, or the broader environment is a vital aspect of early drug development. Traditionally, toxicity evaluation relies on animal testing, which is often time-consuming, costly, and ethically challenging. As an alternative, computational toxicity prediction offers a faster and more resource-efficient approach, eliminating the need for animal models during preliminary screening. In this study, in silico tools such as admetSAR 2.0 and ProTox-II were employed to evaluate the toxicological profiles of the selected compounds. These platforms provided predictions on hepatotoxicity, carcinogenicity, immunotoxicity, mutagenicity, and cytotoxicity, using rat-based models. The results are summarized in Table 4.

Table 4 outlines the toxicity assessment of the compounds, focusing on critical indicators such as hepatotoxicity, carcinogenicity, immunotoxicity, mutagenicity, and cytotoxicity. Compound CID 21820889 demonstrated a favorable safety profile, showing no signs of toxicity across all tested parameters; it was classified as inactive for hepatotoxic, immunotoxic, mutagenic, and cytotoxic responses and exhibited no carcinogenic potential. In contrast, CID 304 showed activity in the immunotoxicity assessment, implying possible immune-related side effects, while remaining non-toxic in terms of hepatotoxicity, mutagenicity, cytotoxicity, and carcinogenicity. These results suggest that CID 21820889 may present a safer therapeutic profile compared to CID 304.

### 3.11. MD Simulation Analysis

To evaluate the stability and dynamic behavior of the protein–ligand complexes, molecular dynamics (MD) simulations were conducted. This computational approach allows for the observation of intermolecular interactions over time, offering detailed insights into the conformational changes of the complexes. A 100-nanosecond MD simulation was carried out to examine the interaction dynamics between the protein and ligand. The resulting simulation trajectories were analyzed to calculate various parameters, including root mean square deviation (RMSD), root mean square fluctuation (RMSF), protein–ligand interaction profiles, and ligand torsion movements. These analyses enhanced our understanding of the structural integrity and binding stability of the complexes throughout the simulation period.

### 3.12. RMSD Analysis

**Figure 6:**
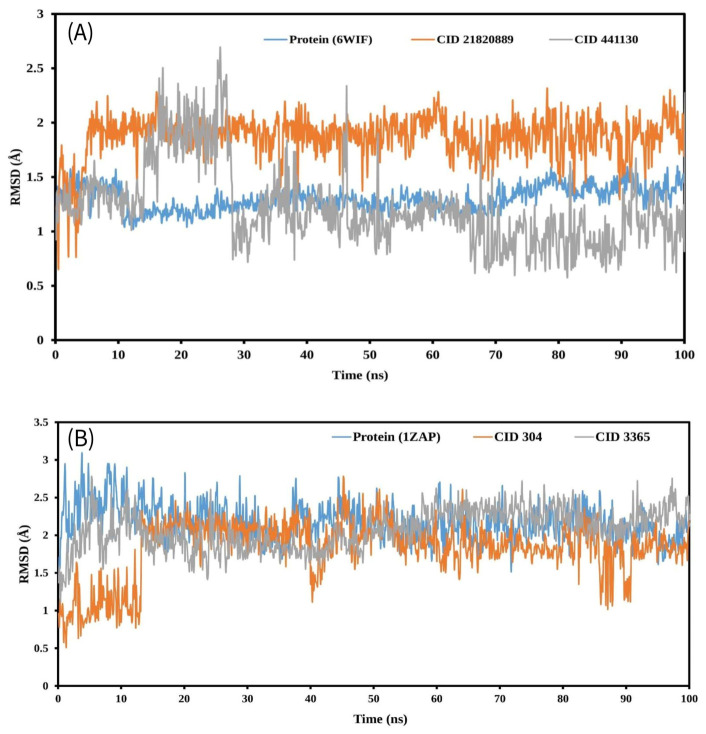
RMSD profiles depicting the stability of ligand–protein complexes during 100 ns molecular dynamics simulations: (**A**) compounds CID 21820889 and CID 441130 with protein 6WIF; (**B**) compounds CID 304 and CID 3365 with protein IZAP. The RMSD trajectories illustrate the convergence behavior and structural fluctuations of each complex, supporting the overall stability of the interactions.

The root mean square deviation (RMSD) values for three ligand molecules and their associated protein complex were analyzed to evaluate the system’s equilibration. In particular, the RMSD profile of compounds CID 21820889 and CID 441130 were compared with the crystal structure of protein 6WIF, as illustrated in Figure 6A, to detect any conformational shifts. Throughout the simulation, RMSD values for the compound ranged from 1.2 Å to 3.5 Å, which falls within an acceptable threshold when aligned with the 6WIF reference structure. Between 30 and 100 nanoseconds, the complex maintained relatively high structural stability. Although some fluctuations were observed during the initial phase of the simulation, the system gradually reached equilibrium. Notably, RMSD values became stable between 70 ns and 100 ns, indicating that the complex had achieved convergence. In a similar analysis, the RMSD values for compounds CID 304 and CID 3365 in complex with the protein IZAP were examined to determine structural stability over time. As shown in Figure 6B, the compound’s RMSD values remained within the range of 1.5 Å to 3.2 Å when benchmarked against the IZAP protein structure. This range was also considered acceptable, suggesting reliable molecular interactions. The most stable period of the simulation occurred between 40 and 100 nanoseconds. While initial movements indicated minor structural adjustments, the system steadily stabilized. Overall, the trajectory demonstrated convergence during the latter half of the simulation, with consistent RMSD values indicating a stable complex formation (Figure 4).

#### 3.12.1. RMSF Analysis

**Figure 7:**
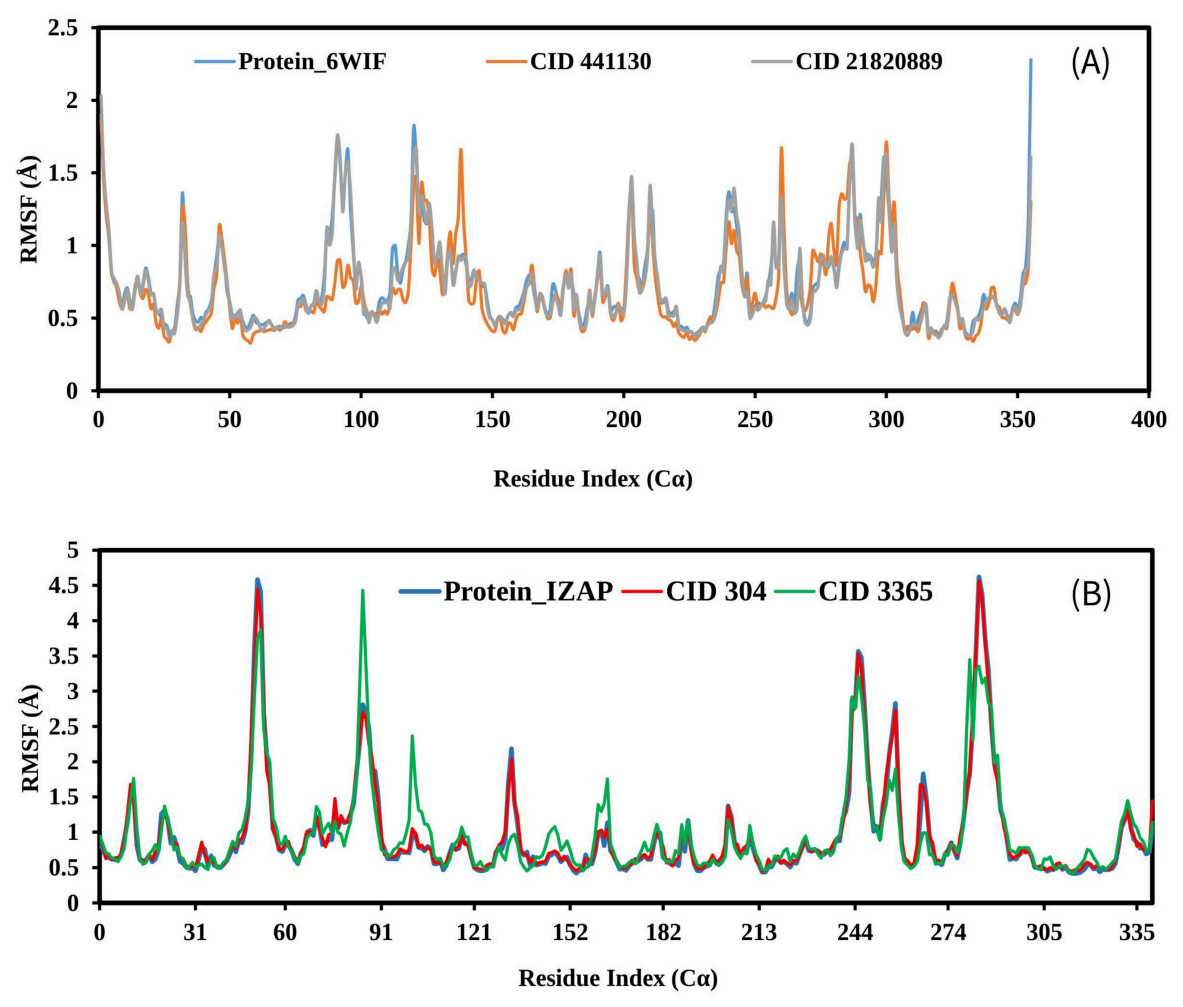
RMSF profiles depicting the stability of ligand–protein complexes during 100 ns molecular dynamics simulations: (**A**) compound CID 21820889 and marketable drug CID 441130 with protein 6WIF; (**B**) compound CID 304 and marketable drug CID 3365 with protein IZAP. The RMSD trajectories illustrate the convergence behavior and structural fluctuations of each complex, supporting the overall stability of the interactions.

To evaluate the flexibility of specific residues during the molecular dynamics simulation, root mean square fluctuation (RMSF) analyses were performed for three compound–protein complexes. These results were then compared against the RMSF profile of the 6WIF protein, as illustrated in Figure 7A, to identify regions with notable atomic fluctuations. The most prominent fluctuation occurred between residue positions 85 and 100, while additional variability was detected between residues 275 and 295, a region associated with the N-terminal domain. Moreover, an elevated RMSF was observed in the C-terminal region, which can be attributed to its surface exposure and reduced secondary structural constraints, allowing for greater conformational flexibility. Overall, the RMSF values for the phytochemically bound complexes demonstrated lower fluctuation levels compared to the unbound 6WIF structure. This indicates that ligand binding contributes to enhanced structural stability of the protein and supports consistent interactions without inducing significant conformational alterations. Complex structures CID 21820889 (Figure 7A) and CID 441130 (Figure 7B) were analyzed with respect to the 100 ns simulation trajectory.

#### 3.12.2. Protein–Ligand Contact Mapping

**Figure 8:**
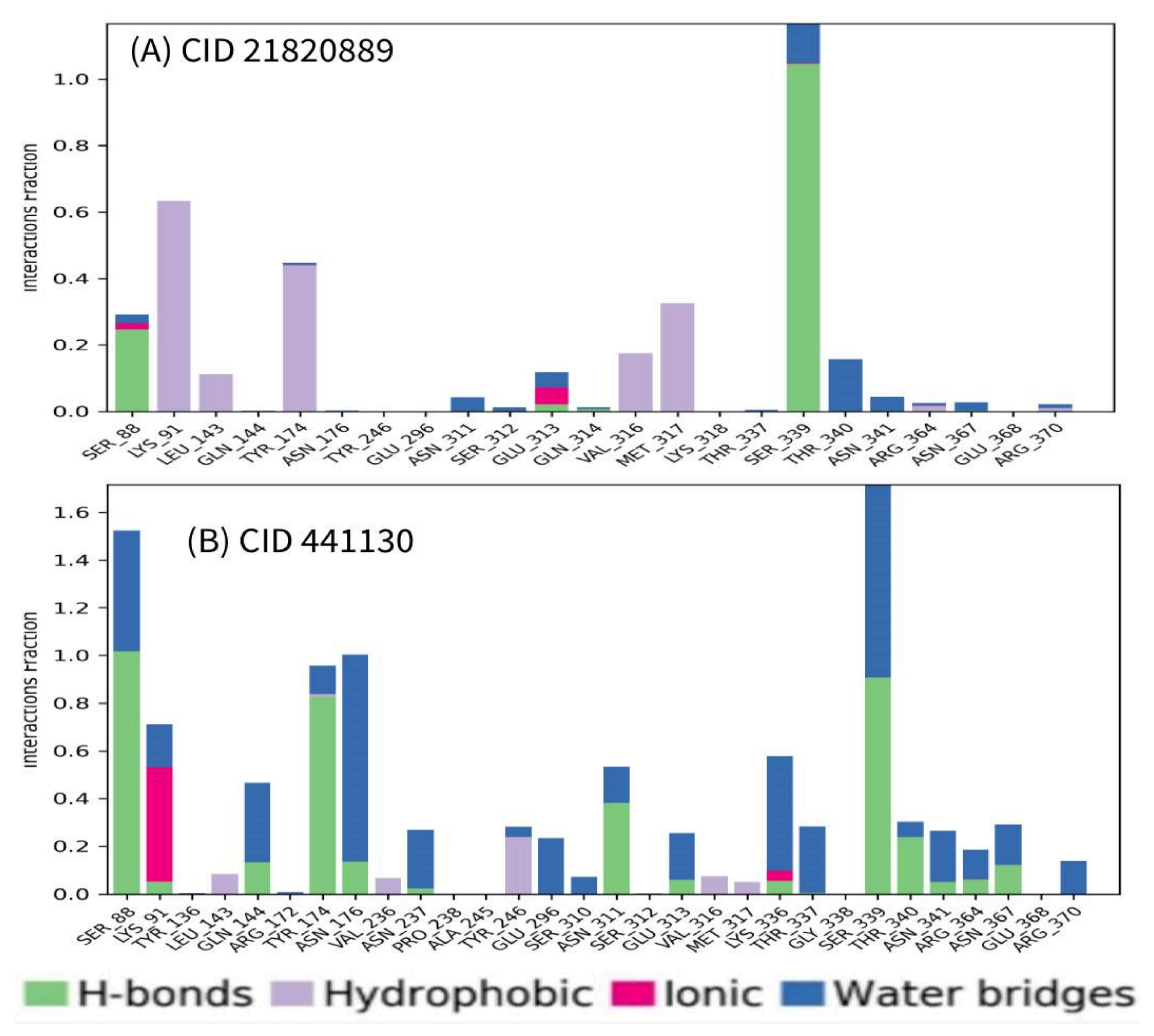
The stacked bar charts representing the contact mapping of protein 6WIF with potential compounds, i.e., (**A**) CID 21820889 and (**B**) CID 441130, extracted from 100 ns simulation trajectories.

**Figure 9:**
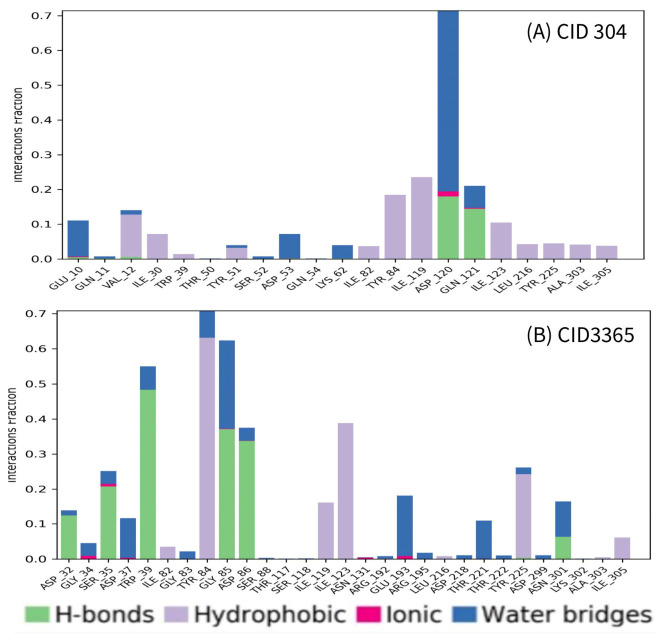
The stacked bar charts representing the contact mapping of protein IZAP with potential compounds, (**A**) CID 304 and (**B**) CID 3365, extracted from 100 ns simulation trajectories.

To gain deeper insight into the molecular interactions between the ligands and their target proteins, protein–ligand contact mapping was performed over the course of the molecular dynamics simulation. During the 100 ns molecular dynamics simulation, the interactions between the 6WIF protein and the selected ligand CID 21820889 and CID 441130 were closely monitored. These interactions were classified into four main types: hydrogen bonding, hydrophobic contacts, ionic interactions, and water bridges. A summary of these interactions is presented as stacked bar charts in Figure 6. The compound demonstrated notable binding affinity through consistent interactions with key catalytic residues within the active site. Over the simulation timeframe, a substantial number of intermolecular contacts were observed, indicating persistent ligand engagement. Active site residues showed frequent interaction with the ligand, and contact density was analyzed for selected candidate compounds. The binding pocket of 6WIF was effectively characterized through natural compound screening and trajectory analysis. Based on these simulation results, CID 21820889 is predicted to form a highly stable complex with 6WIF under physiological conditions, supporting its potential as a strong inhibitor that compared to marketable compound CID 441130 (Figure 8A,B). Similarly, throughout the 100 ns molecular dynamics simulation, the interaction profiles between the IZAP protein and the ligands CID 304 and CID 3365 were systematically examined (Figure 9A,B). The contacts were categorized into hydrogen bonds, hydrophobic interactions, ionic bonds, and water-mediated bridges. These interactions are summarized in the stacked bar graph in Figure 7. The ligand exhibited a strong and consistent binding pattern, particularly with crucial catalytic residues located within the protein’s active site. Over the course of the simulation, the complex maintained a high frequency of intermolecular contacts, reflecting stable ligand engagement. Contact density data were compiled for each of the three compounds analyzed, highlighting the consistent involvement of key active site residues. The ligand-binding region of IZAP was well-defined through virtual screening and trajectory-based analysis. These findings suggest that CID 304 has the potential to maintain a stable and effective binding with IZAP under physiological conditions, reinforcing its promise as a candidate for further biological validation.

### 3.13. Solvent Accessible Surface Area (SASA)

**Figure 10:**
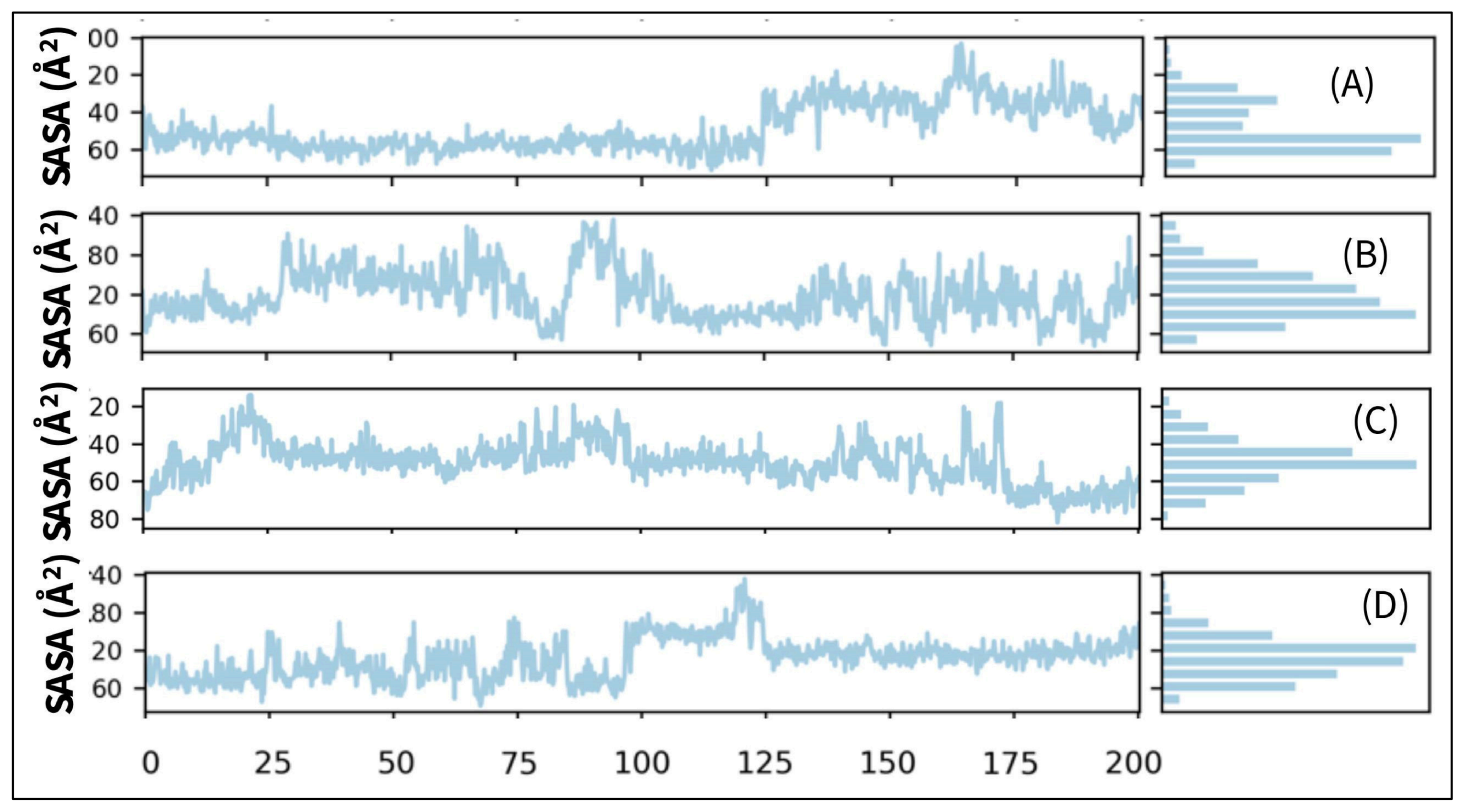
Solvent accessible surface area (SASA) profiles of protein–ligand complexes during molecular dynamics simulations. Time-dependent SASA trajectories are shown for (**A**) CID 21820889, (**B**) CID 441130, (**C**) CID 304, and (**D**) CID 3365. Histograms on the right illustrate the distribution of SASA values sampled throughout the simulations.

The complex containing CID 21820889 exhibited relatively stable SASA values during the first approximately 90 ns, fluctuating around 50–60 Å^2^. After approximately 130 ns, the SASA decreased and remained between approximately 30 and 45 Å^2^ for the remainder of the simulation. The accompanying histogram showed a relatively narrow distribution centered around intermediate SASA values, indicating that the complex sampled a limited conformational space. The CID 441130 complex displayed substantially greater SASA fluctuations throughout the simulation. Multiple increases and decreases were observed between approximately 40 and 70 Å^2^, with frequent transitions between conformational states (Figure 10A). The broader histogram confirmed increased structural heterogeneity and greater solvent accessibility compared with the other complexes.

The CID 304 complex maintained moderate SASA values throughout most of the simulation with fluctuations mainly between 40 and 60 Å^2^. Short-lived decreases in SASA were observed during the early simulation period, followed by relatively stable behavior (Figure 10B). The histogram demonstrated a dominant population near the average SASA, suggesting moderate conformational flexibility. The CID 3365 complex remained relatively stable during the initial simulation period, followed by a transient decrease in SASA around 100 ns, after which the trajectory returned to a stable plateau (Figure 10C). The histogram exhibited a narrow distribution, indicating that the protein largely maintained a compact structural conformation with limited fluctuations. Overall, among the four ligands, CID 21820889 and CID 3365 showed comparatively stable SASA profiles, whereas CID 441130 exhibited the highest solvent accessibility fluctuations (Figure 10D). CID 304 displayed intermediate behavior.

### 3.14. MM/GBSA Binding Free Energy Analysis

**Figure 11:**
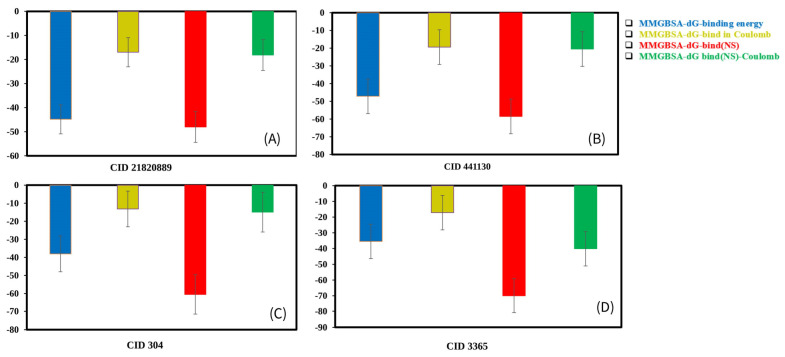
MM/GBSA binding free-energy analysis of the selected protein–ligand complexes following 100 ns molecular dynamics simulations. (**A**) MM/GBSA energy profile of the protein–CID 21820889 complex. (**B**) MM/GBSA energy profile of the protein–CID 441130 complex. (**C**) MM/GBSA energy profile of the protein–CID 304 complex. (**D**) MM/GBSA energy profile of the protein–CID 3365 complex.

The MM/GBSA binding free energy analysis was performed to further evaluate the stability and predicted binding affinity of the selected compounds following molecular dynamics simulations (Figure 11). Overall binding free energies (MMGBSA ΔG-binding energy) ranged from −35.46 to −47.17 kcal/mol (Table S2). Among the investigated compounds, CID 441130 exhibited the most favorable overall binding free energy (−47.17 kcal/mol) (Figure 11B), followed by CID 21820889 (−44.85 kcal/mol) (Figure 11A), CID 304 (−38.09 kcal/mol) (Figure 11C), and CID 3365 (−35.46 kcal/mol) (Figure 11D). The electrostatic (Coulombic) contribution showed relatively similar values among the compounds, ranging from −13.22 to −19.43 kcal/mol. CID 441130 demonstrated the strongest electrostatic interaction (−19.43 kcal/mol), whereas CID 304 showed the weakest Coulombic contribution (−13.22 kcal/mol).

Analysis of the non-solvated interaction energy (MMGBSA ΔG-bind (NS)) revealed more pronounced differences between compounds. CID 3365 exhibited the most favorable non-solvated interaction energy (−70.01 kcal/mol), followed by CID 304 (−60.55 kcal/mol), CID 441130 (−58.51 kcal/mol), and CID 21820889 (−48.02 kcal/mol). Similarly, the non-solvated Coulombic contribution varied substantially among the compounds. CID 3365 displayed the strongest interaction (−40.13 kcal/mol), whereas CID 304 (−14.95 kcal/mol) and CID 21820889 (−18.12 kcal/mol) showed comparatively weaker contributions. Overall, the MM/GBSA analysis suggests that all four compounds formed energetically favorable complexes with their respective target proteins, although differences in the relative energetic contributions indicate distinct interaction mechanisms among the ligands (Figure 11).

## 4. Discussion

The current study investigates the antimicrobial potential of two sea cucumber-derived compounds, CID 21820889 and CID 304, against *A. baumannii* and *C. albicans*, respectively. The study results are consistent with a growing body of research that highlights the bioactive properties of marine-derived compounds, especially those from sea cucumbers, and their potential as therapeutic agents. Sea cucumbers have long been recognized for their diverse bioactive compounds, which include saponins, glycosides, alkaloids, and sterols, all of which exhibit antimicrobial, anti-inflammatory, and anticancer properties [34]. Several studies have demonstrated the antimicrobial potential of sea cucumber compounds against a variety of pathogens, including both bacteria and fungi. Isolated saponins from *Apostichopus japonicus*, a species of sea cucumber, which exhibited significant antibacterial activity against Pseudomonas aeruginosa, Staphylococcus aureus, and Escherichia coli [35]. This is consistent with our findings, where sea cucumber-derived compounds showed specific activity against pathogenic bacteria and fungi [36].

In particular, this study identifies CID 21820889 as an effective compound against *A. baumannii*, a Gram-negative bacterium known for its multidrug resistance. This finding is consistent with previous studies reporting that marine organisms, including sea cucumbers, are rich sources of bioactive metabolites with antibacterial potential [37]. The ability of CID 21820889 to inhibit the growth of this pathogen is of particular importance given the increasing prevalence of drug-resistant bacteria and the urgent need for new therapeutic options.

Similarly, CID 304 demonstrated antifungal activity against *C. albicans*, which corroborates findings from other studies. Marine-derived compounds, particularly from H. scabra, another sea cucumber species, showed significant antifungal activity against *C. albicans*. Our results align with these findings, as CID 304 exhibited strong inhibitory effects against this fungal pathogen. The antifungal potential of marine compounds is becoming increasingly recognized, with several studies investigating the ability of sea cucumber extracts to treat fungal infections, especially in immunocompromised patients [38].

Molecular docking is a widely used computational approach for predicting the binding affinity and interaction patterns of small molecules with biological targets, providing valuable insights into their potential therapeutic efficacy. In the present study, sixteen bioactive compounds identified from the ethanolic extract of *Holothuria scabra* were screened against the antimicrobial target proteins of *Acinetobacter baumannii* (PDB ID: 6WIF) and *Candida albicans* (PDB ID: 1ZAP). The docking analysis revealed considerable variation in binding affinities, suggesting differences in the ability of these metabolites to interact with the active sites of the target proteins. In addition to antimicrobial testing, molecular docking simulations are widely used to predict the binding affinity and interactions between drug candidates and their target proteins. Several studies have employed molecular docking to explore the interactions of marine-derived compounds with bacterial and fungal proteins [39]. Their findings, like ours, highlighted the importance of hydrogen and hydrophobic bonding in the binding process, which contributes to the overall stability of the protein–ligand complex [40]. In this study, CID 21820889 formed hydrogen bonds with the amino acid residues ASN176 and TYR245, along with a hydrophobic interaction involving ASP242. This finding is consistent with previous molecular docking studies that have shown that marine-derived compounds often bind to bacterial targets through similar mechanisms, including hydrogen bonds and hydrophobic interactions. The study reported that marine compounds exhibit such interactions with proteins involved in bacterial cell wall biosynthesis, leading to their antimicrobial action [41].

Similarly, for CID 304, the interactions with the target protein from *C. albicans* were characterized by both hydrogen and hydrophobic bonds. CID 21820889 interacted with Lys87, Tyr105, Asp210, and Arg254, which largely overlapped with the binding residues of the reference drug, indicating a similar binding orientation within the active site. Likewise, CID 304 formed hydrogen bonds with His377, Tyr132, and Ser378, residues also involved in binding the reference antifungal compound, suggesting a comparable inhibitory mechanism. This finding is aligned with studies that demonstrated the binding affinity of marine-derived compounds to fungal proteins, noting that the efficacy of such compounds is often attributed to their ability to form stable complexes with key enzymes in the fungal cell, thus inhibiting fungal growth. Our docking studies further support this hypothesis, indicating that CID 304 may act through similar mechanisms in C. *albicans* [42].

Pharmacokinetic properties play a crucial role in determining the oral bioavailability and therapeutic potential of drug candidates. In our study, both CID 21820889 and CID 304 demonstrated favorable lipophilicity and solubility profiles, which are essential for their ability to cross biological membranes and reach their target sites. The analysis of the pharmacokinetic properties of CID 21820889 and CID 304 is consistent with other studies that have examined marine-derived compounds. The pharmacokinetics of compounds isolated from marine organisms exhibit moderate lipophilicity, which enhances their absorption and distribution in the body. This property is important for the compounds to exert their antimicrobial effects effectively [41].

The ADME analysis of the lead compounds revealed that both candidates violate one or more parameters of Lipinski’s Rule of Five, suggesting potential limitations in oral bioavailability. While these violations may pose challenges for traditional oral administration, they do not necessarily preclude the compounds’ biological activity. Alternative delivery strategies, such as intravenous administration, nanoparticle-mediated delivery, and prodrug formulations, could be employed to enhance systemic availability. Additionally, rational structural modifications of the lead compounds could be explored in future studies to optimize pharmacokinetic properties while preserving or enhancing their antimicrobial efficacy. These considerations highlight the importance of integrating ADME predictions with experimental validation in the early stages of drug development [43]. For example, the development of marine-based anticancer agents has seen a rise in bioavailability-enhancing formulations, such as the use of liposomes or nanoparticles for drug delivery. Therefore, while CID 21820889 and CID 304 may present challenges in terms of oral bioavailability, these challenges can be overcome with formulation strategies that enhance absorption.

The safety profile of a drug candidate is a critical consideration in the early stages of drug development. In silico toxicity prediction, as demonstrated in our study, provides valuable insights into the potential adverse effects of compounds without the need for animal testing. The toxicity profiles of CID 21820889 and CID 304 revealed that CID 21820889 was free from hepatotoxicity, mutagenicity, and cytotoxicity, which aligns with other studies on marine-derived compounds. For example, the certain sea cucumber saponins exhibit low toxicity, suggesting their potential for use in drug development [44].

On the other hand, CID 304 exhibited potential immunotoxicity, which warrants further investigation. While CID 304 showed promising antifungal activity, its potential to affect the immune system raises concerns, particularly for use in immunocompromised individuals. This finding is consistent with studies on other marine compounds, who observed immunotoxicity in certain marine-derived compounds, emphasizing the need for comprehensive toxicity screening before clinical application [45]. Future studies on CID 304 should focus on understanding the mechanisms of immunotoxicity and identifying ways to mitigate these effects while preserving its antifungal efficacy.

SASA analysis indicated distinct differences in solvent accessibility and structural stability among the four protein–ligand complexes. CID 21820889 exhibited a stable SASA profile followed by a gradual decrease after ~130 ns, suggesting increased structural compaction and enhanced protein stability. In contrast, CID 441130 showed pronounced SASA fluctuations throughout the simulation, indicating greater conformational flexibility and comparatively lower stability. CID 304 maintained moderate fluctuations, reflecting stable ligand binding with limited conformational adaptation. CID 3365 remained largely compact, with only a transient conformational change around 100–125 ns before rapidly returning to equilibrium. Overall, the SASA results suggest that CID 21820889 and CID 3365 formed the most compact and structurally stable protein–ligand complexes, whereas CID 304 displayed intermediate stability and CID 441130 exhibited the greatest structural flexibility. These findings are consistent with complementary MD analyses, including RMSD, RMSF, radius of gyration (Rg), hydrogen-bond occupancy, and binding free energy calculations.

The MM/GBSA calculations were conducted to complement the molecular docking and molecular dynamics analyses by providing an estimate of the relative binding free energies of the investigated protein–ligand complexes. These generally provide a more reliable assessment of binding stability than docking scores alone because MM/GBSA incorporates energetic information from MD trajectories. Among the evaluated compounds, CID 441130 exhibited the most favorable overall binding free energy (−47.17 kcal/mol), suggesting a relatively stronger predicted binding affinity toward its target protein. CID 21820889 also demonstrated a favorable binding energy (−44.85 kcal/mol), supporting the stable interactions observed during the molecular dynamics simulations. Although CID 304 and CID 3365 displayed comparatively less favorable total MM/GBSA binding energies, both compounds showed markedly stronger non-solvated interaction energies, particularly CID 3365, which exhibited the lowest MMGBSA ΔG-bind (NS) value (−70.01 kcal/mol). This observation indicates that van der Waals and other non-solvated interactions may contribute substantially to the stabilization of these complexes despite their relatively weaker overall binding energies. Similarly, the Coulombic energy components demonstrated that electrostatic interactions contributed to complex stabilization for all compounds, although the magnitude of these contributions differed among ligands. The relatively balanced distribution of electrostatic and non-solvated energy components suggests that multiple intermolecular forces collectively govern ligand recognition and stabilization within the protein-binding pocket.

The in vitro results of this study, in which CID 21820889 showed activity against *A. baumannii* and CID 304 exhibited effectiveness against *C. albicans*, were corroborated by similar studies in the literature. The marine-derived compounds exhibited strong antimicrobial properties against *A. baumannii*, and compounds from sea cucumbers possessed antifungal activity against *C. albicans* [46]. Our study contributes to this growing body of evidence by further validating the antimicrobial properties of CID 21820889 and CID 304 through the well diffusion method. While the results of this study are promising, it is important to note that in vivo testing is still required to fully assess the therapeutic potential of these compounds. Preclinical animal models are important for evaluating the in vivo efficacy and safety of marine-derived compounds. Therefore, further in vivo studies are necessary to confirm the potential of CID 21820889 and CID 304 as effective therapeutic agents for treating infections caused by *A. baumannii* and *C. albicans*.

## 5. Conclusions

The present study demonstrates that the crude ethanolic extract of *H. scabra* possesses promising antibacterial and antifungal activities, highlighting its potential as a source of marine-derived antimicrobial metabolites. GC–MS analysis tentatively identified sixteen metabolites, among which CID 21820889 and CID 304 were computationally prioritized based on their favorable molecular docking, molecular dynamics simulation, MM/GBSA binding free energy, and ADME prediction results. The MM/GBSA analysis further supported the stability and favorable binding energetics of the predicted protein–ligand complexes, complementing the molecular docking and molecular dynamics findings. Nevertheless, these computational results represent predictive evidence only and should not be interpreted as experimental validation of the biological activities or mechanisms of action of the individual compounds. Overall, the integrated workflow combining GC–MS metabolite profiling, in vitro antimicrobial evaluation of the crude extract, molecular docking, molecular dynamics simulations, MM/GBSA binding free energy calculations, and ADME prediction provides a robust strategy for prioritizing putatively bioactive marine natural products for further investigation. Future studies should focus on bioassay-guided fractionation, purification, structural confirmation using orthogonal analytical techniques such as HRMS and NMR, and direct biological evaluation of the purified compounds to identify the active constituents and validate their antimicrobial potential for therapeutic development.

## Figures and Tables

**Table 1:** Small molecules extracted from the *H. scabra* through the GC-MS.

SL No	Peak Time	Area%	Compound Name	CID
1	3.07	0.18	tetraethyl silicate	CID 6517
2	5.38	0.86	okhotoside B1	CID 44448207
3	11.71	1.34	acetic acid, heptyl ester	CID 8159
4	19.90	0.61	2,4-di-tert-butylphenol	CID 7311
6	25.64	0.62	1,2,3-trimethoxybenzene	CID 12462
5	26.47	7.14	1-methylcyclohexane-1,2,3,4,5,6-hexol	CID 244581
6	27.38	0.73	methyl 10,13-dimethyltetradecanoate	CID 554145
7	28.45	1.21	hexadecanoic acid	CID 985
8	30.95	0.87	17-methyloctadec-13-enoic acid	CID 75955130
9	32.61	5.92	benzeneacetic acid, alpha-methoxy-, (+/−)-	CID 7690
10	34.61	3.40	ergosta-5,22-dien-3.beta.-ol	CID 6432458
11	34.99	6.67	oxiran-2-ylmethyl hexadecanoate	CID 347736
12	36.28	16.11	3-benzyl-4-imino-2-phenylchromeno[3,4-c]pyridin-5-one	CID 21820889
13	36.62	20.03	cholest-5-en-3-ol	CID 304
14	37.51	10.30	methyl 11-methyl-dodecanoate	CID 4065233
15	38.52	8.07	1-methyl-2-nonylcyclohexane	CID 91700840
16	38.82	5.59	heptacosanoic acid, 25-methyl-, methyl ester	CID 554101

**Table 2:** Docking scores of nineteen compounds documented from the sea cucumber.

Compounds and *A. baumannii* (PDB ID: 6WIF)		Compounds and *C. albicans* (PDB ID: 1ZAP)	
CID (Ligand)	Binding Affinity	CID (Ligand)	Binding Affinity
21820889	−8.2	304	−8.4
6432458	−7.9	985	−5.3
304	−6.7	7311	−6.4
244581	−5.9	8159	−4.4
75955130	−5.9	12462	−4.8
554145	−5.6	107202	−5.4
347736	−5.5	244581	−5.8
554101	−5.5	347736	−5
985	−5.4	554101	−5.1
107202	−5.3	554145	−5.1
7311	−5.1	4065233	−5.3
4065233	−5.1	6432458	−8.3
1700840	−4.9	21820889	−7.7
8159	−4.5	44448207	−5.2
12462	−4.4	75955130	−5.4
44448207	−4.1	91700840	−5.3
Marketable Drug (441130)		Marketable drug (3365)	−7.7

**Table 3:** Pharmacokinetic properties of the selected compounds based on ADME analysis.

Properties	Parameters	CID 21820889	CID 304
	MW (g/mol)	378.42 g/mol	386.65 g/mol
	Heavy atoms	29	28
	Arom. heavy atoms	26	0
	Rotatable bonds	3	5
	H-bond acceptors	3	1
	H-bond donors	1	1
	Molar refractivity	115.59	123.61
**Lipophilicity**	Log Po/w	4.94	7.39
**Water solubility**	Log S (ESOL)	−5.47	−7.40
**Pharmacokinetics**	GI absorption	High	Low
**Drug likeness**	Lipinski, violation	Yes	Yes
**Medi. chemistry**	Synth. accessibility	3.32	5.98
**Fathead minnow LC_50_ (96 h)**	mg/L	0.08	0.11
**48 h Daphnia magna LC_50_**	mg/L	0.42	0.60
**Developmental toxicity**	Value	1.00	0.97
**Oral rat LD_50_**	mg/kg	556.02	1078.24
**Bioaccumulation factor**	Log10	1.02	2.87
**Mutagenicity**	Value	Negative	Negative

**Table 4:** The list of the drug-induced toxicity profile includes hepatotoxicity, carcinogenicity, immunotoxicity, mutagenicity, and cytotoxicity of selected compounds.

PubChem ID	Hepatotoxicity	Carcinogenicity	Immunotoxicity	Mutagenicity	Cytotoxicity
21820889	Inactive	No	Inactive	Inactive	Inactive
6432458	Inactive	No	Active	Inactive	Inactive
304	Inactive	No	Lightly active	Inactive	Inactive
244581	Inactive	No	Inactive	Inactive	Inactive
75955130	Inactive	No	Inactive	Inactive	Inactive
554145	Inactive	No	Inactive	Inactive	Inactive
347736	Inactive	No	Inactive	Inactive	Inactive
554101	Inactive	No	Inactive	Inactive	Inactive
985	Inactive	No	Inactive	Inactive	Inactive
107202	Inactive	No	Inactive	Inactive	Inactive
7311	Inactive	No	Inactive	Inactive	Inactive
4065233	Inactive	No	Inactive	Inactive	Inactive
1700840	Inactive	Yes	Active	Active	Active
8159	Inactive	No	Inactive	Inactive	Inactive
12462	Inactive	No	Inactive	Inactive	Inactive
44448207	Inactive	No	Inactive	Inactive	Inactive
Marketable Drug (441130)	Inactive	No	Inactive	Inactive	Lightly Active

## Data Availability

The data presented in this study are available within the article and its Supplementary Materials. Additional data are available from the corresponding author upon reasonable request.

## Supplementary Materials

The following supporting information can be downloaded at https://www.mdpi.com/article/10.3390/biology15151259/s1: Figure S1. The receptor validation and 3D visualization of 1ZAP (**A**) and 6WIF (**B**). Figure S2. The displaying figure showing the frequency of compounds from the sea Cucumber (*Holothuria scabra*). Figure S3A. Ramachandran plot of the 1ZAP protein showing the distribution of backbone φ (phi) and ψ (psi) dihedral angles. Most residues are located within the favored and additionally allowed regions, indicating good stereochemical quality and structural reliability. Figure S3B. Ramachandran plot of the 6WIF protein showing that the majority of residues occupy the favored β-sheet and right-handed α-helical regions, with only a few outliers. This confirms the good stereochemical quality and structural integrity of the protein model. Table S1. Structural validation metrics of the target proteins used for molecular docking. Table S2. MM/GBSA binding free energy components of the selected protein–ligand complexes calculated from the 100 ns molecular dynamics simulation trajectories. Table S3. Two-way ANOVA and Tukey’s post-hoc analysis of inhibition zone diameters (mm) of the *Holothuria scabra* ethanolic extract against *A. baumannii* and *C. albicans*.

## Abbreviations

The following abbreviations are used in this manuscript:

ADME	Absorption, distribution, metabolism and excretion
MD	Molecular dynamics simulation
RMSD	Root mean square deviation
RMSF	Root mean square fluctuation
CADD	Computer-aided drug design
PDB	Protein data bank
