# Supplementary material for: Integrated Molecular Docking and Molecular Dynamics Approaches Reveal Potential Antimicrobial Metabolites from Echinoderms, Holothuria scabra
Source: Biology (Basel). 2026 Jul 31;15(15):1259. doi: 10.3390/biology15151259 (PMC13465092; doi:10.3390/biology15151259)
Supplement: Supplementary file 1 [file biology-15-01259-s001.zip › Supplementary_Material_1.pdf]

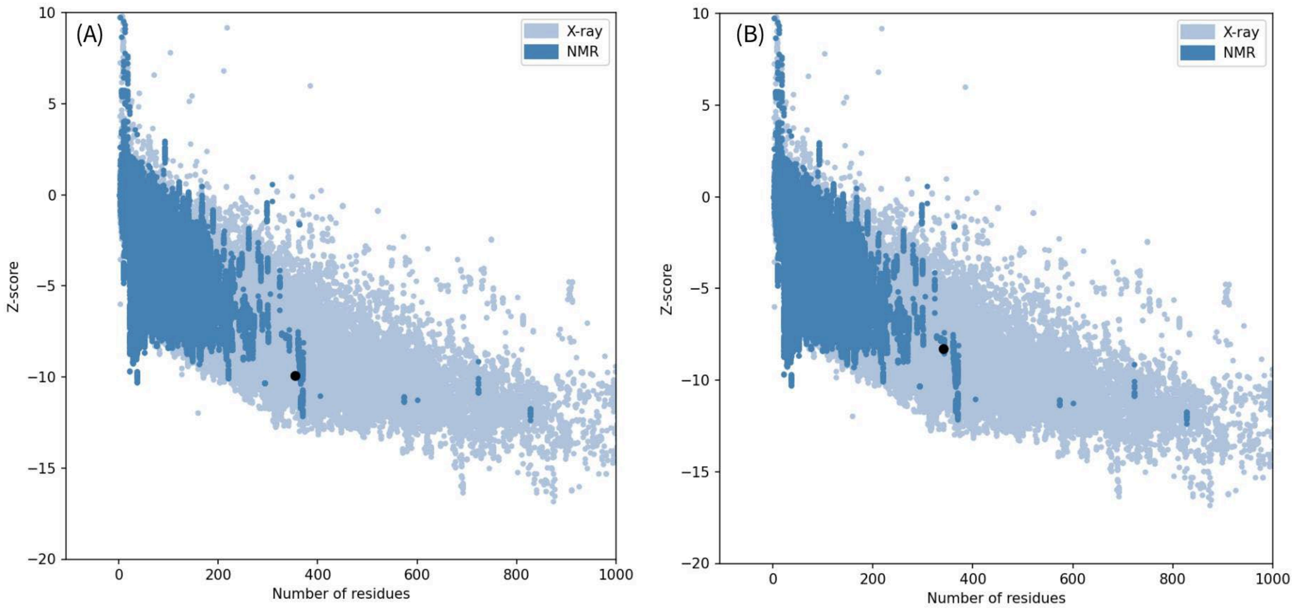

Figure S1. The receptor validation and 3D visualization of 1ZAP (A) and 6WIF (B).

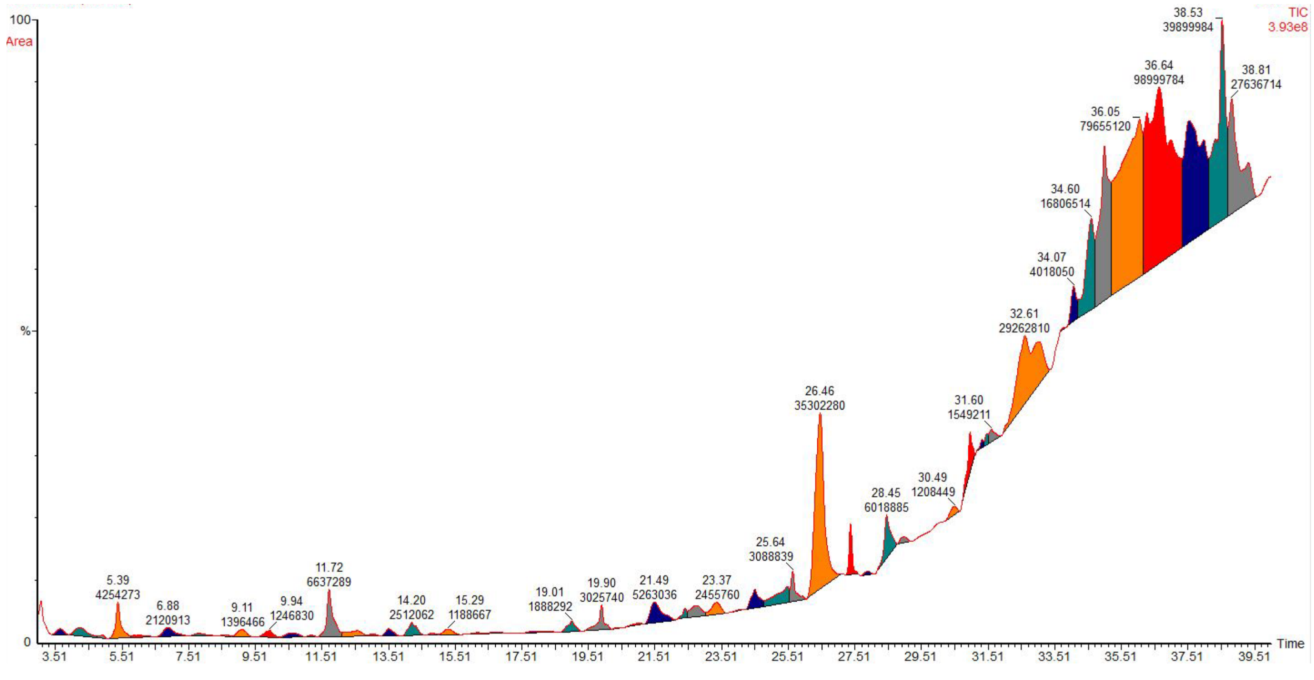

Figure S2. The displaying figure showing the frequency of compounds from the sea Cucumber (*Holothuria scabra*).

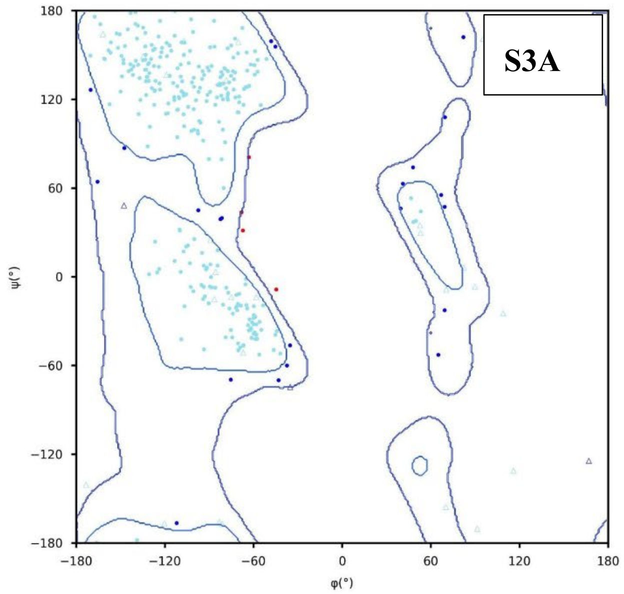

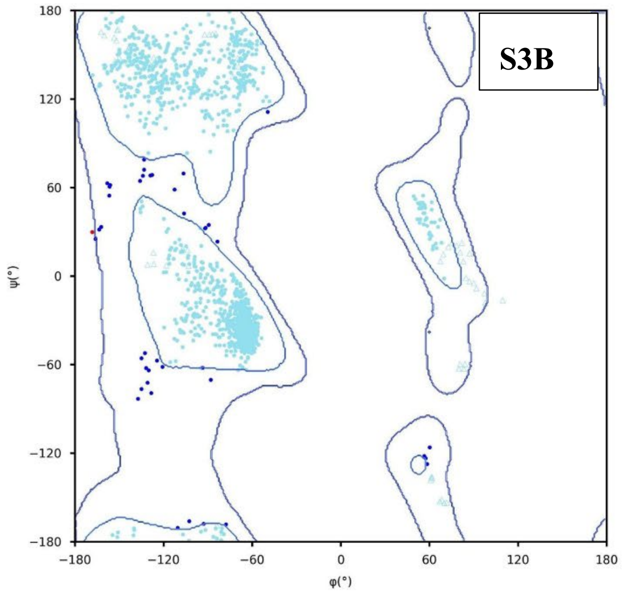

Figure S3A. Ramachandran plot of the 1ZAP protein showing the distribution of backbone φ (phi) and ψ (psi) dihedral angles. Most residues are located within the favored and additionally allowed regions, indicating good stereochemical quality and structural reliability. **Figure S3B.** Ramachandran plot of the 6WIF protein showing that the majority of residues occupy the favored β-sheet and right-handed α-helical regions, with only a few outliers. This confirms the good stereochemical quality and structural integrity of the protein model.

Table S1. Structural validation metrics of the target proteins used for molecular docking.

PDB ID	Organism	Resolution (Å)	Z-score (ProSA)	Residues in Favored Region (%)	Additional Allowed (%)	Generously Allowed (%)	Disallowed (%)
1ZAP	Candida albicans	2.50 Å	-8.3	-91.45%		-7.38%	-1.18%
6WIF	Acinetobacter baumannii	2.15 Å	-9.92	-97.04%		-2.89%	-0.07%

Table S2: MM/GBSA binding free energy components of the selected protein–ligand complexes calculated from the 100 ns molecular dynamics simulation trajectories.

**Compound**	**MMGBSA-dG-binding energy**	**MMGBSA-dG-bind in Coulomb**	**MMGBSA-dG-bind(NS)**	**MMGBSA-dG bind(NS)-Coulomb**
CID21820889	-44.8499	-16.9768	-48.0216	-18.1245
CID 441130	-47.1706	-19.4305	-58.5148	-20.5074
CID304	-38.0858	-13.2166	-60.5522	-14.9453
CID 3365	-35.4586	-17.181	-70.0087	-40.1273
