# Supplementary material for: Integrated Molecular Docking and Molecular Dynamics Approaches Reveal Potential Antimicrobial Metabolites from Echinoderms, Holothuria scabra
Source: Biology (Basel). 2026 Jul 31;15(15):1259. doi: 10.3390/biology15151259 (PMC13465092; doi:10.3390/biology15151259)
Supplement: Supplementary file 1 [file biology-15-01259-s001.zip › Table S3. Two-way ANOVA and Tukey's post-hoc analysis of inhibition zone diameters (mm) of the Holothuria scabra ethanolic extract against A. baumannii and C. albicans..pdf]

Two-Way ANOVA: Tukey

Run at: 5 /7 /2026`Â #£##£ , E

Descriptive Statistics

Cell (Pathogen : Species)	N	Mean	SD	SEM
0.5 mg/ml : A. baumannii	3	9.0000	1.0000	0.5774
0.5 mg/ml : C. albicans	3	11.6667	0.5774	0.3333
1 mg/ml : A. baumannii	3	11.0000	1.0000	0.5774
1 mg/ml : C. albicans	3	20.6667	1.5275	0.8819

Two-Way ANOVA Results

Source	Sum of Squares	Mean Square	df	F	P-value	Significant
Pathogen	90.7500	90.7500	(1, 8)	77.7857	<0.0001	Yes
Species	114.0833	114.0833	(1, 8)	97.7857	<0.0001	Yes
Pathogen × Species	36.7500	36.7500	(1, 8)	31.5000	0.000503	Yes
Residual	9.3333	1.1667	8	—	—	—
Total	250.9167	—	11	—	—	—

Effect Size (Eta-squared)

Source	eta²
(Intercept)	0.9955
Pathogen	0.9067
Species	0.9244
Pathogen × Species	0.7975

Tukey Post-Hoc Comparisons

Comparison	Difference	P-value (adj)	95% CI	Significant
0.5 mg/ml A. baumannii - 1 mg/ml A. baumannii	-2.0000	0.185032	[-4.8242, 0.8242]	No
0.5 mg/ml A. baumannii - 0.5 mg/ml C. albicans	-2.6667	0.064323	[-5.4909, 0.1575]	No
0.5 mg/ml A. baumannii - 1 mg/ml C. albicans	-11.6667	<0.0001	[-14.4909, -8.8425]	Yes
1 mg/ml A. baumannii - 0.5 mg/ml C. albicans	-0.6667	0.871756	[-3.4909, 2.1575]	No
1 mg/ml A. baumannii - 1 mg/ml C. albicans	-9.6667	<0.0001	[-12.4909, -6.8425]	Yes
0.5 mg/ml C. albicans - 1 mg/ml C. albicans	-9.0000	<0.0001	[-11.8242, -6.1758]	Yes

Shapiro-Wilk Normality Test

Cell	W Statistic	P-value
0.5 mg/ml : A. baumannii	1.0000	1.0000
0.5 mg/ml : C. albicans	0.7500	0.0000
1 mg/ml : A. baumannii	1.0000	1.0000
1 mg/ml : C. albicans	0.9643	0.6369

Levene's Test of Equal Variance

DF1	DF2	F Statistic	P-value
3	8	0.4444	0.7278
